# Host ecotype and rearing environment are the main drivers of threespine stickleback gut microbiota diversity in a naturalistic experiment

**DOI:** 10.1098/rsos.240649

**Published:** 2024-06-26

**Authors:** Andreas Härer, Christine J. Frazier, Diana J. Rennison

**Affiliations:** ^1^ School of Biological Sciences, Department of Ecology, Behavior & Evolution, University of California San Diego, La Jolla, CA, USA

**Keywords:** gut microbiome, threespine stickleback, *Gasterosteus aculeatus*, 16S rRNA sequencing, host–microbiota interaction, vertebrate

## Abstract

Host–microbiota interactions play a critical role in the hosts’ biology, and thus, it is crucial to elucidate the mechanisms that shape gut microbial communities. We leveraged threespine stickleback fish (*Gasterosteus aculeatus*) as a model system to investigate the contribution of host and environmental factors to gut microbiota variation. These fish offer a unique opportunity for experiments in naturalistic conditions; we reared benthic and limnetic ecotypes from three different lakes in experimental ponds, allowing us to assess the relative effects of shared environment (pond), geographic origin (lake-of-origin), trophic ecology and genetics (ecotype) and biological sex on gut microbiota α- and β-diversity. Host ecotype had the strongest influence on α-diversity, with benthic fish exhibiting higher diversity than limnetic fish, followed by the rearing environment. β-diversity was primarily shaped by rearing environment, followed by host ecotype, indicating that environmental factors play a crucial role in determining gut microbiota composition. Furthermore, numerous bacterial orders were differentially abundant across ponds, underlining the substantial contribution of environmental factors to gut microbiota variation. Our study illustrates the complex interplay between environmental and host ecological or genetic factors in shaping the stickleback gut microbiota and highlights the value of experiments conducted under naturalistic conditions for understanding gut microbiota dynamics.

## 1. Introduction

Interactions between host organisms and the complex microbial community residing in their guts (i.e. the gut microbiota) affect many aspects of the hosts’ biology [1,2]. At the same time, variation in host-associated and environmental factors shapes gut microbiota composition. Important host factors include genetics, diet, age and physiology [3–6], whereas key environmental factors include temperature, salinity, geographic and seasonal variation or exposure to pollutants [7–13]. Quantifying their relative contributions to gut microbiota variation can provide crucial insights into the mechanisms that shape microbial communities and their functional roles within the host but is often challenging, especially in wild populations where it is difficult to account for or measure these factors. In contrast, studies performed under controlled laboratory conditions can help isolate the contribution of environmental or host factors to microbiota variation. However, in the lab, the gut microbiota is commonly altered by the highly artificial conditions, and results might therefore not translate to wild populations [14,15]. Thus, it can be difficult to determine the most biologically relevant factors structuring microbiota variation. One particularly promising way to make progress that avoids these limitations and allows quantification of the major determinants of gut microbiota variation is to perform experiments under naturalistic conditions.

Threespine stickleback fish (*Gasterosteus aculeatus*, hereafter ‘stickleback’) are highly suitable for naturalistic experiments and are an emerging model system in microbiota research [16–22]. Initial studies have begun to explore the influence of host ecology, genotype, diet, geographic distance and habitat type on gut microbial diversity. A study across co-occurring threespine stickleback and ninespine stickleback (*Pungitius pungitius*), two species that diverged around 26 million years ago [23], found that a larger proportion of gut microbiota variation was explained by habitat type than by host species identity [24]. Variation in the gut microbiota of a wild stickleback population has been shown to be influenced by diet diversity, sex and the immune system of the host [16,25,26]. Across wild stickleback populations, there is evidence that variation in the gut microbial communities of populations from estuarine and freshwater habitats can be explained by host genetic divergence and habitat type [20,21], and that gut microbiota divergence between freshwater populations that differ in their trophic ecology is, to some extent, predictable [19]. Freshwater threespine stickleback populations have diverged in their trophic ecology, feeding on two types of prey associated with different habitats: littoral invertebrates from the lake sediment (benthic prey) or pelagic zooplankton (limnetic prey) [27]. Besides this divergence in trophic ecology, benthic and limnetic ecotypes differ in various aspects, including microhabitat use, morphology (including body size and shape, mouth width, gill raker number and length) and genetics [27–31]. Notably, benthic and limnetic ecotypes evolved repeatedly and independently [30,32], thus providing a powerful system for studying gut microbiota changes across ecologically divergent but closely related host populations. In summary, variation in the gut microbiota within and across stickleback populations appears to be shaped by several ecological and evolutionary processes. However, no prior work has been able to comprehensively disentangle the relative contributions of these various factors to patterns of gut microbial diversity. Given the ability to perform experiments conducted in naturalistic conditions, it is possible to quantify the effects of these factors on the stickleback gut microbiota.

**Figure 1.**
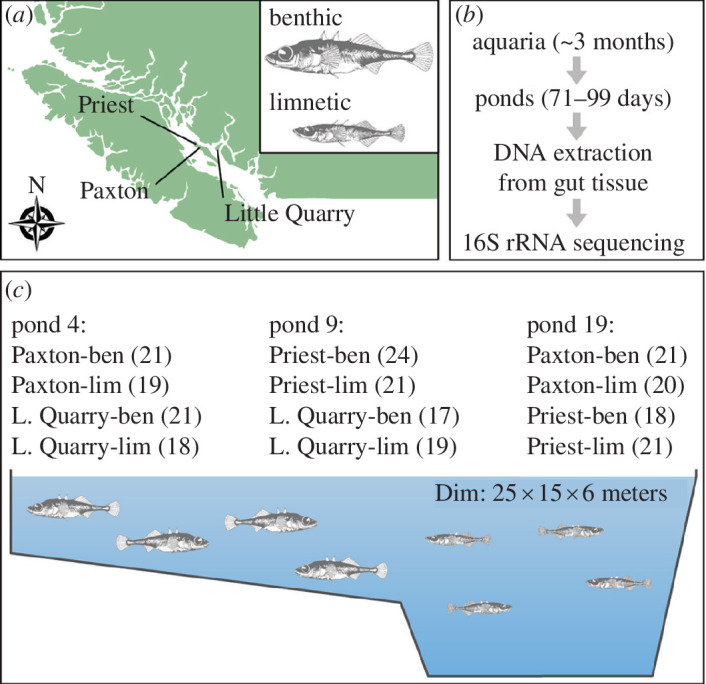
The benthic and limnetic ecotypes used in our experiment originate from three lakes in British Columbia, Canada: Paxton Lake, Priest Lake and Little Quarry Lake (*a*). At an age of approximately 3 months, fish were introduced into experimental ponds for 71–99 days (*b*). Each experimental pond contained a combination of benthic and limnetic populations from two different lakes and sample sizes ranged from 17 to 21 fish (ben: benthic, lim: limnetic) (*c*). The ponds contain shallow littoral and deeper open-water zones that are preferentially occupied by benthic and limnetic ecotypes, respectively (*c*).

Here, we used a large-scale experimental infrastructure to assess structuring of the stickleback gut microbiota. To this end, benthic and limnetic ecotypes from three lakes in British Columbia, Canada, were reared in three experimental ponds in different pairwise combinations (figure 1). This setting allowed us to tease apart the relative contributions of a shared environment (pond), host geographic origin (lake-of-origin), host trophic ecology and genetics (ecotype) and host sex in shaping the composition and diversity of stickleback gut microbiota. We specifically tested for effects of the aforementioned factors on gut microbiota α- and β-diversity, as well as the taxonomic composition on the level of bacterial orders. We predicted gut microbiota composition to be more similar among hosts of the same pond, ecotype, lake-of-origin and biological sex, but we refrained from making specific predictions about their relative contributions since these factors have been shown to collectively shape gut microbial communities and their importance can vary across study systems.

## 2. Material and methods

### 2.1. Experimental design

The fish included in this study represent a subset of the fish used in an experiment that investigated the fitness consequences of hybridization (note that no hybrids were used for our study), and we refer to the original study for more detailed methodological information [33]. Experimental fish were obtained from within-population crosses of benthic and limnetic ecotypes from Paxton Lake, Priest Lake and Little Quarry Lake in British Columbia, Canada. Until the start of the experiment, the parental fish and experimental fish were kept under common conditions in aquaria and provided with a standardized diet. When experimental fish reached the juvenile stage, they were introduced into three semi-natural ponds at the University of British Columbia, Canada. The ponds have dimensions of 25 × 15 m and have both benthic and limnetic habitats, allowing fish to occupy and forage in different microhabitats according to their natural behaviour [31]. Each experimental pond contained a combination of benthic and limnetic populations from two different lakes (i.e. a total of four populations per pond) (figure 1). The experimental fish were roughly of the same age (between 2.5 and 3.5 months) when introduced into the ponds, and the experiment lasted for a timespan ranging from 71 to 99 days. Wild progenitors of the fish used in our experiment were collected between 2017 and 2019 under the following permit numbers SU17-258923, SARA17-PPAC-00002, MRSU18-288855, SARA18-PPAC-00006, MRSU19-454239 and SARA19-PPAC-00006 issued by Fisheries and Oceans Canada and the Ministry of Forests, Lands, Natural Resource Operations. The experiment was conducted in accordance with institutional guidelines under the animal care permit numbers A16-0044 and A20-0050 approved by the UBC Animal Care Committee.

### 2.2. Data collection

At the end of the experiment, fish were euthanized with an overdose of MS-222 and stored at −20°C. Our dataset consisted of a total of 240 fish that were equally distributed across ponds, ecotypes and lakes. Sample sizes for populations within each pond ranged from 17 to 24 (electronic supplementary material, table S1). To determine sex, we extracted DNA from fin tissue and used a PCR assay developed by Peichel *et al*. [34]. Fish specimens were rinsed with 95% ethanol, and whole guts were dissected using sterile equipment. To minimize the contribution of transient bacteria, we carefully removed gut contents and the guts were then stored at −80°C until DNA extraction. We extracted DNA from whole guts using the QIAGEN PowerSoil Pro Kit (Qiagen, Hilden, Germany) according to the manufacturer’s protocol under sterile conditions in a laminar flow hood. To amplify the V4 region of the 16S rRNA gene, we used barcoded 515F and 806R primers obtained from https://github.com/SchlossLab/MiSeq_WetLab_SOP/blob/master/MiSeq_WetLab_SOP.md. PCR reactions were performed in triplicate using the Q5 High-Fidelity 2X Master Mix (New England Biolabs, Ipswich, MA, USA), with replicates pooled after amplification. The PCR protocol consisted of a denaturation step at 98°C for 60 s, 35 amplification cycles with 10 s at 98°C, 20 s at 56°C and 60 s at 72°C, followed by a final elongation at 72°C for 10 min. PCR products were visualized using gel electrophoresis on a 2% agarose gel to confirm successful amplification, and DNA concentrations were measured using a Qubit 4 Fluorometer (Thermo Fisher Scientific, Waltham, MA, USA). Negative controls of sterile water were included for DNA extraction and PCR, and no detectable DNA amplification was observed. Subsequently, all samples were equimolarly pooled to create two libraries (samples were sequenced either in 2021 or 2022). Samples were randomly distributed between libraries in regard to pond, lake-of-origin, ecotype and sex (electronic supplementary material, table S1). The libraries were purified using bead clean-up at the UC Davis Genome Center, and DNA quality was assessed using a Bioanalyzer (Agilent Technologies, Santa Clara, CA, USA). The final libraries were sequenced on the Illumina MiSeq 600 (PE300) platform.

To gather information regarding sticklebacks’ diet, muscle tissue was sampled for the assessment of stable isotope ratios of carbon (δ^13^C) and nitrogen (δ^15^N). These ratios enable the identification of diet variation associated with benthic and limnetic habitats [35,36]. Muscle tissues were dried at 55°C and subsequently homogenized, 1 mg of each sample was loaded into a tin capsule and combusted in a Elementar vario EL cube elemental analyzer interfaced to an Elementar VisION IRMS (Elementar Analysensysteme GmbH, Germany) at the UC Davis Stable Isotope Facility. Laboratory standards indicated measurement errors (s.d.) of ±0.05‰ for δ^13^C and ±0.07‰ for δ^15^N.

### 2.3. Data analysis

We obtained a total of 9 493 085 raw sequencing reads, with an average of 39 554 reads per sample. Because of low sequencing depths in some samples, and further reduction during the merging of forward and reverse reads, we decided to use 250 bp of the forward reads for our gut microbiota analyses. Forward reads consistently exhibited superior sequence quality compared with the reverse reads and covered 86% of the target locus (250 out of 291 bp). All upstream analyses were performed in QIIME2 [37]. We used the dada2 plugin to obtain amplicon sequencing variants (ASVs) [38], constructed a bacterial phylogenetic tree with FastTree 2.1.3 [39] and assigned taxonomy based on the SILVA 138 ribosomal RNA (rRNA) database with a 99% similarity threshold [40]. We further excluded ASVs with less than 10 reads that were detected in only one sample, ASVs that could not be classified at the class level, and ASVs associated with chloroplasts, mitochondria, cyanobacteria or archaea to focus solely on the bacterial gut microbiota. Scaling with ranked subsampling (SRS) was used to normalize ASV counts with a *C*_min_ of 2500 reads [41]. We determined the proportion of unique and shared ASVs based on pond, lake-of-origin, ecotype and sex.

We studied the relative contributions of pond, lake-of-origin, ecotype, sex and carbon and nitrogen isotope signatures to variation in gut microbiota α-diversity (ASV richness, Faith’s phylogenetic diversity, Shannon diversity) and β-diversity (non-phylogenetic: Bray–Curtis dissimilarity, phylogenetic: unweighted and weighted UniFrac; [42,43]). We further tested whether sequencing library (sequenced either in 2021 or 2022) affected α- and β-diversity. For α-diversity, we used linear models (*lm* function in stats package v. 4.2.1) [44] and type III ANOVA (*Anova* function in car package v. 3.1-0) [45], followed by pairwise Wilcoxon rank-sum tests (*wilcox.test* function in stats package v. 4.2.1) with Bonferroni correction of *p* values. When diet (based on carbon and nitrogen isotope signatures) and ecotype were both included in a model, there were no significant residual effects of ecotype after accounting for diet effects. This suggests that ecotype effects on α-diversity are primarily driven by differences in diet and niche use rather than due to other co-varying factors such as differences in physiology (including immunology), behaviour or life history. Given the statistical equivalency of isotope signature (diet) and ecotype, we proceeded with the analysis using ecotype alone for simplicity. We did not observe any effect of sequencing library on α-diversity, and model outcomes did not qualitatively differ for the other independent variables depending on whether sequencing library was included or not.

**Figure 2.**
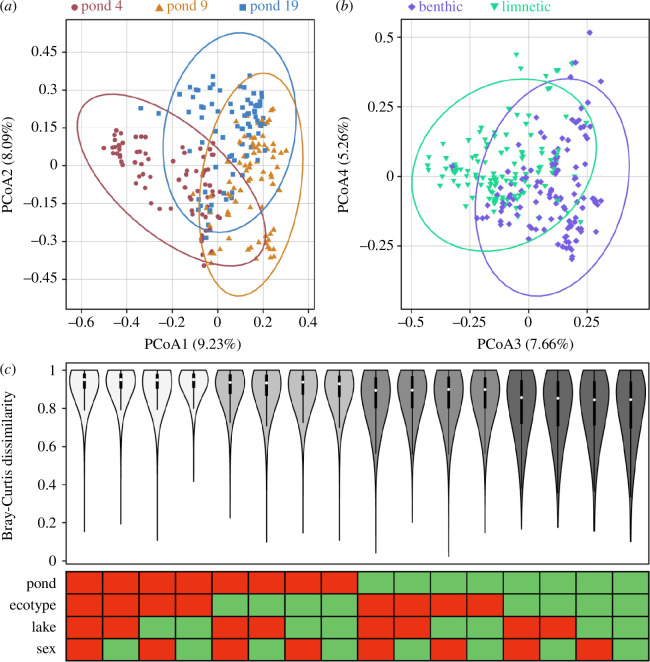
Principal coordinate analysis based on Bray–Curtis dissimilarity showed evidence for divergence among populations from the three experimental ponds along PCoA axes one and two (*a*) as well as divergence among ecotypes along PCoA axes three and four (*b*). Pairwise comparisons of Bray–Curtis dissimilarity values were calculated among all individuals that belonged to the same (green boxes) or a different (red boxes) group (*c*). For example, the first column only contains comparisons among individuals that belonged to the same pond, ecotype, lake-of-origin and sex, whereas the last column only contains comparisons among individuals that belonged to different groups. β-diversity was mostly affected by pond and host ecotype, as indicated by the decrease in Bray–Curtis dissimilarity associated with it (highlighted by differently shaded groups of violin plots).

For β-diversity, we used PERMANOVA (*adonis2* function in vegan package v. 2.6-2) [46,47] with the *by = ‘margin’* option to test for marginal effects of the aforementioned factors on gut microbiota dissimilarity. We detected weak, but significant, effects of sequencing library but again incorporating sequencing library did not affect model outcomes for the other independent variables. We visualized changes in β-diversity values depending on whether comparisons were made between hosts from the same or different pond, ecotype, lake-of-origin and sex (figure 2*c*). Additionally, we used Mantel tests to test for correlations between β-diversity and divergence in genetics and body shape on the host population level. Genetic divergence was assessed based on *F*_ST_ values that were obtained from [48], note that these represent wild individuals that were not included in our study. For all fish included in our study, we determined body shape based on geometric morphometric analysis using 17 digital landmarks [49] (electronic supplementary material, figure S1). We took photographs of the left lateral side of each specimen under standardized conditions including a ruler for scale. Landmarking was done in tpsDig2 [50]. Data were then imported into morphoJ [51] for Procrustes fitting, outlier removal and producing a Procrustes distance matrix across stickleback populations. To investigate differences in within-population β-diversity, we calculated the distance of each fish from its population centroid and statistically tested for differences among groups (*betadisper* function in vegan package v. 2.6-2). Differential relative abundance of bacterial orders and genera between ponds, host ecotypes, lakes-of-origin and sexes was assessed by analysis of composition of microbiomes (ANCOM) [52]. We visualized taxonomic composition of the gut microbiota on the bacterial order level, but only included orders that on average comprised more than 1% of the bacterial community in any of the host populations. All statistical analyses were done in R v. 4.2.1 [44].

## 3. Results

### 3.1. Variation in gut microbiota α-diversity is mostly explained by host ecotype and pond

**Figure 3.**
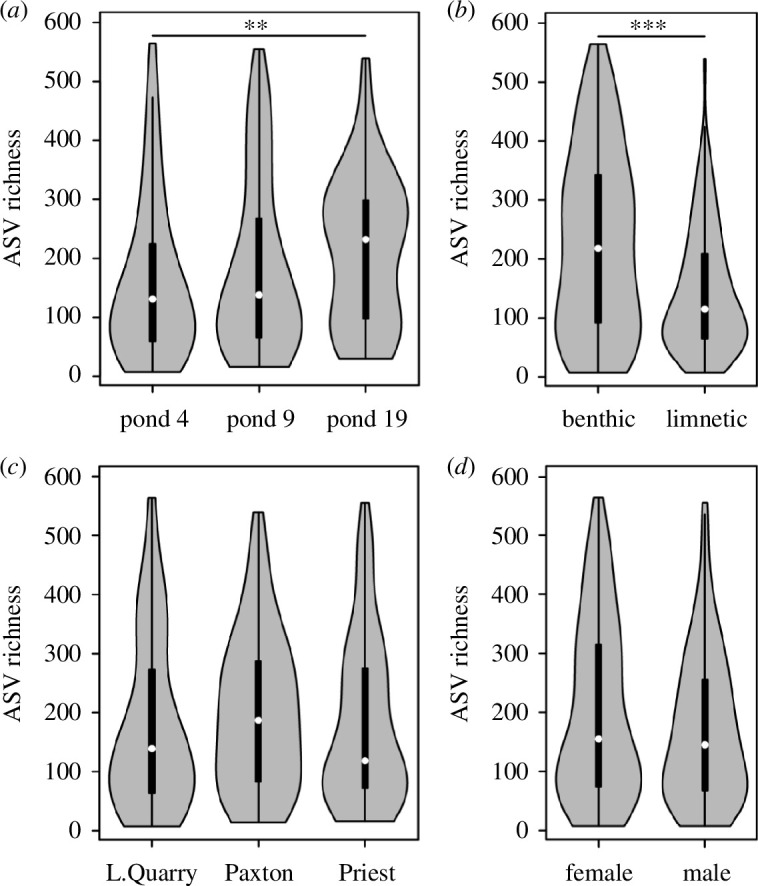
Bacterial α-diversity (ASV richness) differed across ponds (*a*) and ecotypes (*b*), but not across lakes-of-origin (*c*) or sexes (*d*) based on linear models (test statistics for all three α-diversity metrics can be found in electronic supplementary material, table S2). Pairwise Wilcoxon rank-sum tests revealed that ASV richness was higher in pond 19 compared to pond 4 (*a*) and also in benthic fish compared to limnetic fish (*b*). **p < 0.01, ***p < 0.001.

There were a total of 6461 ASVs across all samples. The major bacterial orders that on average constituted more than 5% of the gut microbiota were Pirellulales (13.87%), Clostridiales (8.76%), Bacillales (7.80%), Holosporales (7.44%), Gemmatales (5.38%) and Izimaplasmatales (5.22%) (electronic supplementary material, figure S2). While many ASVs were shared across ponds, lakes-of-origin, ecotypes and sexes, we found a relatively equal proportion of unique ASVs based on these groupings (electronic supplementary material, figure S3). For example, 1915 and 1699 ASVs were exclusively found in benthic and limnetic fish, whereas 2847 ASVs were found in both ecotypes. ASV richness was primarily affected by host ecotype (linear model; *F*_1,239_ = 23.06, *p* < 0.001) and to a lesser extent by pond (*F*_2,239_ = 4.34, *p* = 0.014), but not significantly affected by lake-of-origin (*F*_2,239_ = 1.42, *p* = 0.243) or host sex (*F*_1,239_ = 0.79, *p* = 0.375) (figure 3). Benthic fish had a 90% higher ASV richness than limnetic fish (median: 218 versus 115) (Wilcoxon rank-sum test; *W* = 9316, *p* < 0.001), and ASV richness was also higher in pond 19 compared to pond 4 (*W* = 2297, *p* = 0.009) (electronic supplementary material, table S2). We found qualitatively similar results for Faith’s phylogenetic diversity (ecotype: *F*_1,239_ = 19.16, *p* < 0.001; pond: *F*_2,239_ = 5.88, *p* = 0.003), but there was only suggestive evidence for an effect of host ecotype on Shannon diversity (*F*_1,239_ = 3.35, *p* = 0.068) (electronic supplementary material, table S2). Higher α-diversity in benthic fish was a largely consistent pattern found when analysing fish from different ponds and lakes-of-origin separately (electronic supplementary material, table S3). In sum, host ecotype was consistently the main driver of variation in α-diversity across three metrics with higher α-diversity in benthic fish, and we further observed a weaker, and inconsistent, effect of pond, which was mainly driven by higher α-diversity in pond 19.

### 3.2. Variation in gut microbiota β-diversity is mostly explained by pond and host ecotype

Pond was the major contributor to explaining gut microbiota β-diversity (PERMANOVA; *R* ^2^ = 0.086, *F*_2,238_ = 12.04, *p* = 0.001) followed by host ecotype (*R* ^2^ = 0.036, *F*_1,238_ = 9.99, *p* = 0.001), with weaker effects of lake-of-origin (*R* ^2^ = 0.010, *F*_2,238_ = 1.35, *p* = 0.035) and host sex (*R* ^2^ = 0.005, *F*_1,238_ = 1.29, *p* = 0.097) based on Bray–Curtis dissimilarity (figure 2*a,b*). Both UniFrac metrics also revealed strong effects of pond and host ecotype, as well as suggestive evidence for an effect of lake-of-origin based on weighted UniFrac (electronic supplementary material, table S4). These results were confirmed by comparisons of β-diversity values calculated between hosts from the same or different pond, ecotype, lake-of-origin and sex which revealed that being from the same pond was the strongest predictor of gut microbiota similarity followed by host ecotype, whereas no apparent effect of lake-of-origin or host sex was observed (figure 2*c*). Next, we incorporated diet data (stable isotope signatures of carbon and nitrogen) into our models to test whether ecotype effects were maintained after accounting for diet. Results were mixed across β-diversity metrics; ecotype still had a significant effect on Bray–Curtis dissimilarity (*R* ^2^ = 0.010, *F*_1,228_ = 2.61, *p* = 0.001) and weighted UniFrac (*R* ^2^ = 0.014, *F*_1,228_ = 3.65, *p* = 0.001), whereas both carbon (Bray–Curtis dissimilarity: *R* ^2^ = 0.007, *F*_1,228_ = 1.89, *p* = 0.003; weighted UniFrac: *R* ^2^ = 0.009, *F*_1,228_ = 2.39, *p* = 0.009) and nitrogen (Bray–Curtis dissimilarity: *R* ^2^ = 0.010, *F*_1,228_ = 2.66, *p* = 0.001; weighted UniFrac: *R* ^2^ = 0.019, *F*_1,228_ = 5.08, *p* = 0.001) signatures were also significant. For unweighted UniFrac, the effect of ecotype (*R* ^2^ = 0.004, *F*_1,228_ = 0.95, *p* = 0.453) was not significant anymore, whereas nitrogen signature (*R* ^2^ = 0.012, *F*_1,228_ = 3.11, *p* = 0.002) showed a significant effect.

Since benthic and limnetic ecotypes from the three lakes have been shown to differ morphologically and genetically, we used geometric morphometric and genetic data to test for associations of these factors with gut microbiota β-diversity. We found evidence for positive correlations between divergence in body shape and gut microbiota β-diversity among populations based on Bray–Curtis dissimilarity (Mantel test, *R* ^2^ = 0.520, *p* = 0.033), unweighted UniFrac (*R* ^2^ = 0.419, *p* = 0.042) and weighted UniFrac (*R* ^2^ = 0.549, *p* = 0.068). At the same time, we detected positive correlations between genetic divergence (F_ST_ values calculated from wild specimens of benthic and limnetic ecotypes from the three lakes) and gut microbiota β-diversity based on Bray–Curtis dissimilarity (Mantel test, *R* = 0.610, *p* = 0.003) and weighted UniFrac (*R* = 0.632, *p* = 0.007).

**Figure 4.**
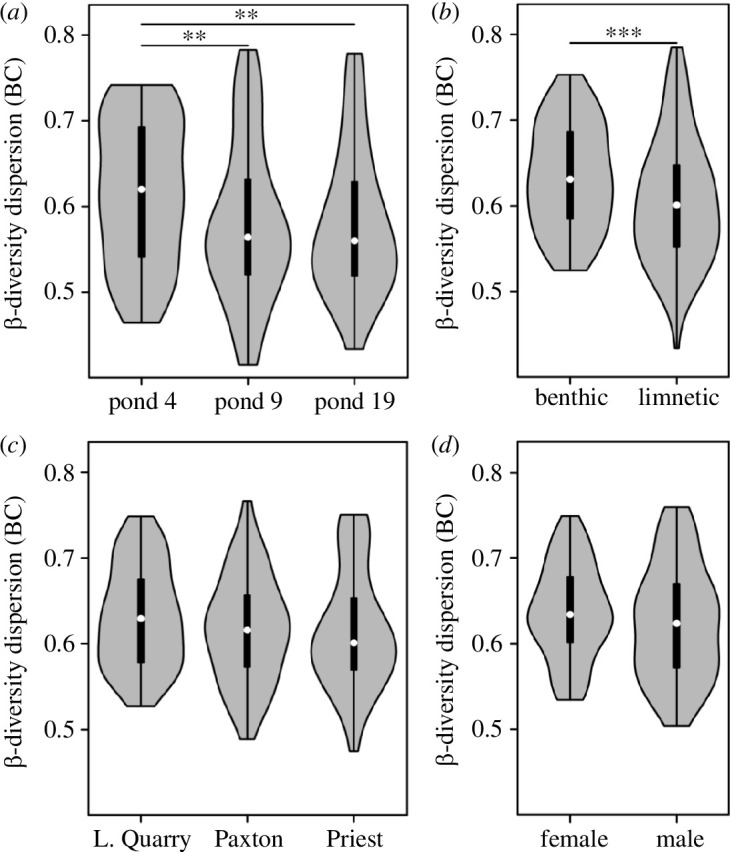
Dispersion of within-group β-diversity (measured as Bray–Curtis distances from group centroid) differed across ponds (*a*) and ecotypes (*b*), but not across lakes-of-origin (*c*) or sexes (*d*) based on PERMDISP (test statistics for all three β-diversity metrics can be found in electronic supplementary material, table S5). Pairwise Wilcoxon rank-sum tests revealed that β-diversity dispersion was higher in pond 4 compared with the other two ponds (*a*) and also in benthic fish compared to limnetic fish (*b*). **p < 0.01, ***p < 0.001.

Next, we tested for differences in within-group β-diversity dispersion across ponds, ecotypes, lakes-of-origin and sex. Within-group β-diversity differed between ponds based on Bray–Curtis dissimilarity (PERMDISP; *R* ^2^ = 0.037, *F*_2,239_ = 4.61, *p* = 0.009) with higher dispersion in pond 4 compared to the other two ponds and unweighted UniFrac (*R* ^2^ = 0.050, *F*_2,239_ = 6.28, *p* = 0.004) with lower dispersion in pond 19 compared to the other two ponds (figure 4*a*, electronic supplementary material, table S5). An effect of host ecotype was only detected for Bray–Curtis dissimilarity (*R* ^2^ = 0.044, *F*_1,239_ = 10.86, *p* = 0.001) where β-diversity dispersion was lower in limnetic fish (figure 4*b*). There was only suggestive evidence for an effect of lake-of-origin based on unweighted UniFrac (*R* ^2^ = 0.024, *F*_2,239_ = 2.87, *p* = 0.056) (figure 4*c*), and none of the metrics revealed differences between host sexes (figure 4*d*).

### 3.3. Most differentially abundant bacterial orders found across ponds

Consistent with β-diversity results, we found the strongest evidence for a pond effect on bacterial community composition as 23 bacterial orders were differentially abundant across ponds (ANCOM; electronic supplementary material, table S6). Among these bacterial orders, the following phyla were most prevalent: Proteobacteria (eight orders), Chloroflexi (seven orders) and Actinobacteria (three orders). In comparison, five bacterial orders were differentially abundant between ecotypes which belonged to the phyla Chloroflexi (three orders), Planctomycetes (one order) and Proteobacteria (one order). Three orders belonging to the phyla Proteobacteria (two orders) and Planctomycetes (one order) were differentially abundant across lakes. Only one bacterial order, the Bifidobacteriales, belonging to the phylum Actinobacteria was differentially abundant between sexes with a higher abundance in females (but relative abundance was very low, 0.1% and 0% in females and males, respectively). We further conducted differential abundance testing for bacterial genera, and results were similar in the sense that the strongest effect was observed across ponds, followed by ecotypes, lakes-of-origin and sexes (electronic supplementary material, table S7).

## 4. Discussion

Which are the major determinants of gut microbiota variation within and across host populations? This intriguing question has been addressed in a broad range of phylogenetically diverse host organisms (e.g. [4,5,53]). Yet, the relative contributions of different host-associated and environmental factors are often unclear and can differ across host species [3,54–57]. Here, we leveraged a unique experimental setting to quantify the relative contributions of three host-associated factors (ecotype, lake-of-origin and sex) and rearing environment (pond) on shaping the stickleback gut microbiota. This setting is exceptional since the experimental ponds are relatively large (25 × 15 m and up to 6 m deep) and contain benthic and limnetic microhabitats (figure 1*c*), which provides stickleback the opportunity to use these distinct microhabitats and feed on associated differential dietary resources [35]. This is supported by the observation that patterns of dietary diversity seen in the experimental ponds are similar to those seen in wild populations [31]. Thus, this experimental setting allows studying the stickleback gut microbiota under naturalistic conditions while controlling for environmental variation.

Benthic and limnetic stickleback ecotypes differ in many aspects of their biology, including morphology, lifespan, habitat use, genetics, and most importantly for our study, diet [27–30]. Accordingly, we found that host ecotype was the major determinant of gut microbiota α-diversity. When accounting for diet (carbon and nitrogen isotope signatures), there was no residual effect of ecotype on α-diversity, strongly suggesting that dietary differences between ecotypes are indeed the main driver of ecotype-associated variation in α-diversity. Moreover, gut morphology and physiology have been shown to affect microbiota diversity [10,58,59], and these factors could also differ between stickleback ecotypes, particularly since benthic and limnetic fish differ not only in diet but also in body size [27]. Gut microbiota α-diversity increases with body size across vertebrates in accordance with the diversity–area relationship [10,58], and thus, the higher α-diversity we found in benthic fish could, to some extent, be explained by differences in body size and gut length between ecotypes. Yet, we currently cannot disentangle the effects of the ecotype-specific aspects mentioned above and future studies should strive to obtain a more mechanistic understanding of the specific factors that produce gut microbiota differences between stickleback ecotypes. Rearing environment also had a significant, albeit weaker, effect, whereas neither lake-of-origin nor host sex affected α-diversity. While we found host ecotype to be the strongest predictor of α-diversity, a previous study comparing threespine stickleback and ninespine stickleback found that environmental differences were the main determinant of α-diversity, even stronger than host species identity [23]. Consistent with our results, a large-scale analysis across 128 mammalian species found that diet was the only significant predictor of α-diversity but no effects of geography, habitat or host phylogeny were observed [5]. Further studies across different animal hosts found similar patterns. For example, experimental diet manipulation showed that α-diversity varies with diet but not with sex, age or habitat type in great tits [60]. In fish, host diet is associated with variation in α-diversity in African cichlids [61], whereas no such patterns were observed between benthic and limnetic ecotypes of whitefish [62] or Neotropical cichlids [63]. In stickleback, a study on benthic and limnetic populations from the same lakes used here found no difference in α-diversity between ecotypes [19]. Yet, it should be noted that the study by Rennison *et al*. investigated wild populations, so confounding factors that were controlled for in our experiment but not in the wild could obscure ecotype-specific gut microbiota patterns. Such factors might include variations in abiotic (e.g. temperature and salinity) and biotic (e.g. prey items and environmental microbes) components of the lake environment [9,13,20]. Furthermore, this study was limited to five individuals per ecotype and lake which might provide another explanation as to why no differences were detected. In contrast, α-diversity has been shown to vary with diet along the benthic–limnetic axis in one stickleback population from Vancouver Island, Canada [16]. Furthermore, while there appears to be some variation in α-diversity across six stickleback populations from Oregon [21], no such pattern was found across 14 populations from Vancouver Island that differ in the proportion of benthic and limnetic diet items as well as diet diversity [17]. In sum, our results are generally in line with work highlighting the importance of host trophic ecology on gut microbiota α-diversity but results in stickleback have been mixed so far. Notably, our findings suggest a link between the recent ecological divergence (<12 000 years) between benthic and limnetic stickleback and changes in gut microbiota α-diversity. However, the functional implications for the host organism and the reasons why benthic stickleback show higher α-diversity remain to be determined but differences in diet diversity between ecotypes or differential exposure to environmental microbes could explain some of this variation [16,20,36].

Similar to α-diversity, rearing environment and host ecotype were the major determinants of β-diversity, but in this case, rearing environment had the strongest effect. This pattern was further supported by the observation that the largest number of differentially abundant bacterial orders and genera was found among ponds followed by host ecotypes (electronic supplementary material, tables S6 and S7). Gut microbiota variation across ponds might be produced by stochastic differences in biological communities; differences in prey items such as zooplankton or invertebrates and the microbial communities of the water could lead to differential exposure to as well as acquisition and selection of microbes [20,64]. Furthermore, gut bacteria can disperse among host individuals leading to changes in gut microbiota composition [65], and homogenization of gut microbial communities among individuals of the same pond might have contributed to the stronger divergence among ponds. Our results strongly suggest that gut microbiota composition is primarily shaped by the environment (and potentially horizontal transfer among host individuals reared in the same environment), which is in line with previous results on co-occurring threespine stickleback and ninespine stickleback [23]. Moreover, abiotic and biotic factors such as temperature and salinity can have strong effects on the gut microbiota [9,13]. We do not expect these factors to vary between our experimental ponds for several reasons; all ponds were filled from the same water source, they were not heated or manipulated in any other way. They contain the same volume of water and are located just metres from each other. Thus, these experimental ponds are exposed to the same climatic conditions at the research facility including ambient temperature and precipitation. Unfortunately, these factors were not measured during our experiment and thus, cannot be explicitly examined. Overall, the strong pond effects on gut microbiota variation, even within this controlled experiment with minimal environmental variation across replicate ponds, is of strong interest for the microbiome research community, and the observed patterns are highly relevant for our biological understanding of the major factors that shape gut microbiota variation in nature. Our results further highlight the need to control for environmental variation (e.g. across replicates in experimental studies) when studying the effects of host-associated factors on gut microbiota variation.

The effect of host ecotype on β-diversity further highlights the importance of diet on the gut microbiota, and this was confirmed by the observation that carbon and nitrogen isotope signatures, two indicators of host diet, had significant effects on the stickleback gut microbiota. Similar patterns of differences in gut microbiota community composition associated with host diet have been described for a large number of animal hosts [5,66,67] including fishes [22,61,63,64,68]. Gut microbiota divergence between benthic and limnetic ecotypes has been shown in whitefish, but results vary across natural replicates [62]. A recent stickleback study further found evidence for gut microbiota divergence across ecotypes collected from the wild but not when reared under laboratory conditions and fed a common diet [19]. In combination with our results, this suggests that the ability for benthic and limnetic ecotypes to occupy and forage in different microhabitats might be necessary for producing detectable gut microbiota divergence. Yet, after accounting for diet, we found that ecotype still had a significant effect on Bray–Curtis dissimilarity and weighted UniFrac (but not on unweighted UniFrac), indicating that other ecotype-associated aspects of the sticklebacks’ biology such as physiology or genetics may also affect gut microbiota composition. The divergence of gut microbial communities between ecotypes might also be produced by different microbial communities associated with microhabitats or food items, and specifically testing for the contribution of these microbial sources could be a focus of future studies. While the fish gut microbiota commonly differs strongly from the bacterioplankton community [62,63], we cannot rule out that this might still affect the stickleback gut microbiota. Studying microbes associated with food items could be particularly interesting since dietary input of microbes has been shown to affect the stickleback gut microbiota [20].

We further detected strong positive correlations between divergence in host body shape and β-diversity, which highlights that body shape might be instrumental to predicting gut microbiota divergence among stickleback fish. Notably, previous genomic work revealed that diet tends to co-map with trophic morphology [69], indicating that diet differences are to some degree heritable. Thus, the correlation between gut microbiota dissimilarity and genetic divergence (*F*_ST_) might be a signature of greater divergence in trophic ecology. Interestingly, we also found that the gut microbiota of limnetic fish were more similar to each other (i.e. lower β-diversity dispersion) compared to benthic fish. Since host genetics and diet are hypothesized to be the major determinants of gut microbiota composition, the observed pattern might be driven by a higher dietary or genetic similarity among limnetic fish. Accordingly, we found that benthic fish showed a higher variance of carbon isotope signatures but not nitrogen isotope signatures. Yet the genetic similarity hypothesis remains to be explicitly tested as this would require individual genotypic data, which we did not have. Potential effects of diet diversity on the gut microbiota are largely unexplored but a previous study across wild stickleback populations found evidence for a positive association between diet diversity and gut microbiota uniqueness [17]. While benthic and limnetic stickleback have been shown to differ in a range of ecologically relevant phenotypes, our results indicate that this phenotypic divergence also extends to the gut microbiota.

Effects of lake-of-origin and host sex on β-diversity were much weaker than other host or environmental factors, and results were inconsistent across metrics. Our results regarding host sex are in line with previous results on wild stickleback populations from Vancouver Island, Canada, for which there was also no effect of host sex on gut microbiota composition [20]. Another study found a significant interaction between diet and host sex, but no main effect of host sex, on the stickleback gut microbiota [25]. Divergence of the stickleback gut microbiota (β-diversity) across wild host populations can be partially explained by genetic divergence among host populations [20,21], raising the question of how this might have affected the patterns observed here. Notably, we did not find strong evidence for effects of lake-of-origin on gut microbiota α- and β-diversity, whereas host ecotype had a much stronger effect. Besides variation in trophic ecology, this pattern could be explained by the fact that genetic divergence across stickleback from the lakes investigated here is generally higher between ecotypes compared to within the same ecotype, and levels of genetic divergence between ecotypes of the same lake are similar to those found within ecotypes of distinct lakes [48,70]. Genetic divergence among host lineages is often positively associated with gut microbiota divergence [21,71,72], and we found evidence for a positive correlation of these measures based on two β-diversity metrics (Bray–Curtis dissimilarity and weighted UniFrac). Thus, parallel genetic divergence of stickleback ecotypes, in combination with divergence in trophic ecology, can be hypothesized to be a major factor shaping gut microbiota variation.

Here, we performed an experiment under naturalistic conditions to investigate the gut microbiota of three pairs of recently and independently diverged stickleback ecotypes. We found that host ecotype had the strongest effect on α-diversity, suggesting that α-diversity is predominantly controlled by variation in host trophic ecology. In contrast, β-diversity was mostly affected by the rearing environment, indicating that the composition of gut microbial communities is largely a product of variation in abiotic and biotic environmental conditions. These results highlight that different aspects of the stickleback gut microbiota are predominantly shaped by different factors and emphasize the importance of considering environmental variation when studying gut microbial communities across host populations from distinct habitats. Stickleback show repeated ecological divergence along the benthic–limnetic axis both in sympatry and in allopatry across many closely related natural replicates (diverged within the last 12 000 years; [27]). This, in combination with the observed association between host ecotype and their gut microbiota composition and diversity, makes stickleback a powerful system to investigate how gut microbiota variation might affect and be affected by local adaptation to distinct ecological niches.

## Data Availability

The raw sequencing data [73] and all associated files and R scripts [74] have been deposited on Figshare. Supplementary material is available online [75].
